# Sudden acquired retinal degeneration syndrome may be an acquired primary ciliopathy, phenotypically similar to human Alström and Bardet-Biedl syndromes

**DOI:** 10.3389/fvets.2025.1611850

**Published:** 2025-06-06

**Authors:** Steven Toler, Kenneth Abrams, Daniel Ward

**Affiliations:** ^1^ClinPharmTox, LLC, Houston, TX, United States; ^2^Veterinary Ophthalmology Services, North Kingstown, RI, United States; ^3^College of Veterinary Medicine, University of Tennessee, Knoxville, TN, United States

**Keywords:** SARDS, retina, systemic symptoms, ciliopathy, vision, canine

## Abstract

Sudden acquired retinal degeneration syndrome (SARDS) is an acquired canine disease that presents as rapidly progressive retinal degeneration, often accompanied by polyphagia, weight gain, polydipsia, polyuria, and hyposmia. Alström syndrome (AS) and Bardet-Biedl syndrome (BBS) are rare human autosomal recessive genetic disorders marked by progressive retinopathy, polyphagia, obesity, polydipsia, polyuria, and hyposmia, with varying degrees of phenotypic severity. While the etiology of AS and BBS is partially understood, the cause of acquired SARDS remains elusive. Historically, scientific inquiry has focused on an immunologic insult and/or endocrinopathy as the cause of SARDS. Clinicians have often pointed to these Cushingoid symptoms mentioned above in SARDS patients as evidence of a contributing endocrinopathy. However, systemic cortisol concentrations, both pre- and post-ACTH stimulation, typically do not differ appreciably between normal patients and those with SARDS. Blindness due to photoreceptor degeneration, along with the observed Cushingoid symptoms, may result from dysfunctional or absent primary cilia, as documented in human AS and BBS cases. Recognizing SARDS as a possible acquired ciliopathy may be the first step toward seeking effective treatments.

## 1 Introduction

Sudden acquired retinal degeneration syndrome (SARDS) is an acquired canine disease that presents as rapidly progressive retinal degeneration, often accompanied by polyphagia, weight gain, polydipsia, polyuria, and hyposmia (1, 2).

Alström syndrome (AS) and Bardet-Biedl syndrome (BBS) are rare human autosomal recessive genetic disorders marked by progressive retinopathy, obesity, polyuria, and hyposmia, with varying degrees of phenotypic severity (3, 4). While these two maladies exhibit many similarities, they are separate diseases with a common cause. AS is caused exclusively by a known defect in the *ALMS1* gene, which encodes a protein involved in the formation and maintenance of primary (non-motile) cilia. BBS results from dysfunctions in over 20 genes, primarily involving those related to cilia development and maintenance, such as *ALMS1* and *BBS1* through *BBS20* (3, 5). Primary cilia are microtubule-based organelles that detect and relay extracellular signals, permitting sensory cells to “translate” or allow adjustments to environmental conditions.

**Figure 1:**
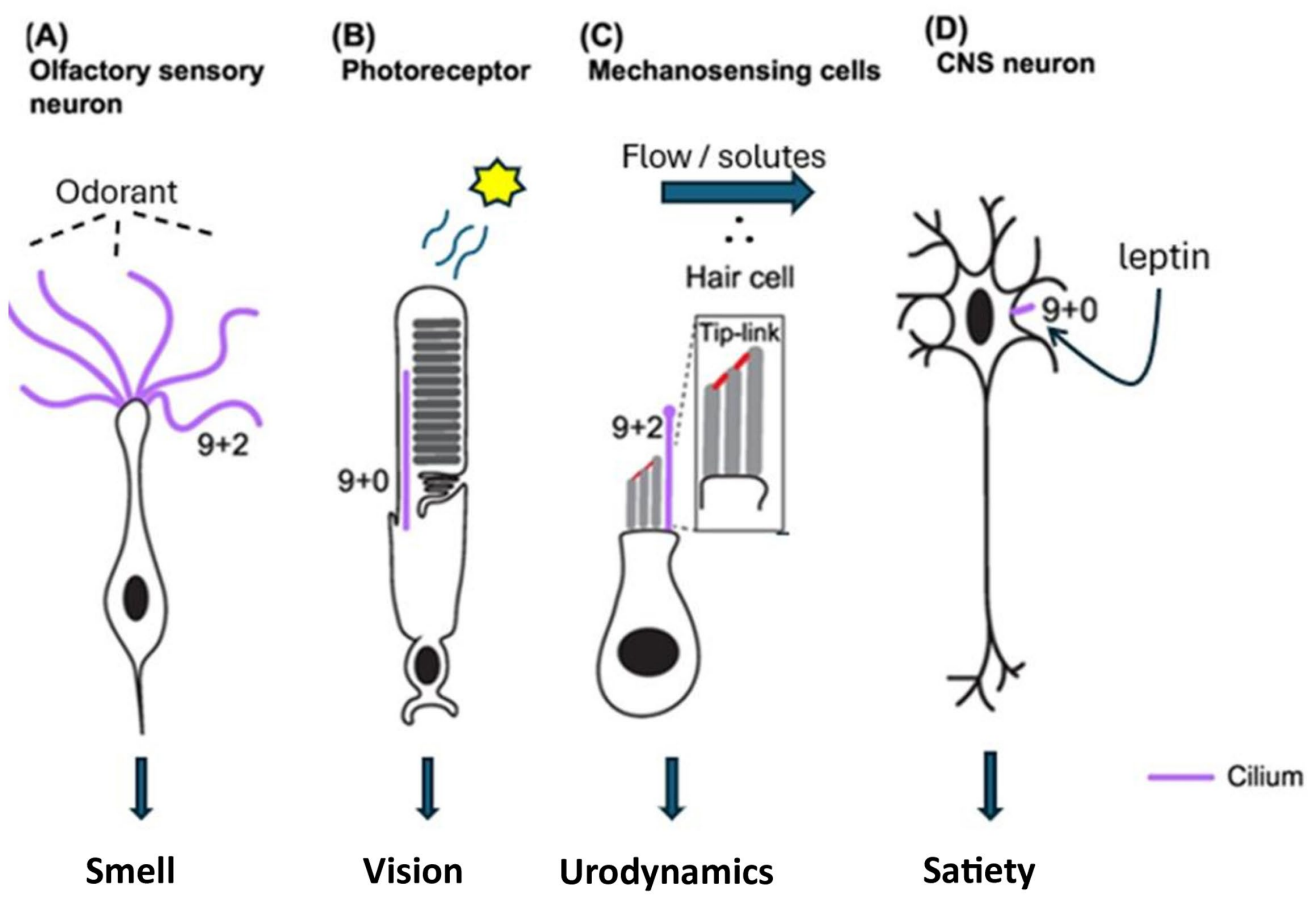
Ciliated neurosensory cells associated with clinical symptoms in BBS, AS, and SARDS [Adapted from Jurisch-Yaksi et al. (33)].

Ciliated cells play a vital role in vision, appetite, renal function, and olfaction (Figure 1). The clinical presentation of SARDS appears to closely resemble that of BBS and AS, syndromes known to be caused by ciliopathies. Visual and systemic symptoms associated with AS, BBS, and SARDS are not reversible and persist indefinitely (6, 7). Therefore, the congruence of target organ pathology, neurosensory deficits, and persistent clinical course noted in BBS, AS, and SARDS suggests that SARDS may also result from ciliary dysfunction.

### 1.1 Vision

Photoreceptor outer segments are specialized primary cilia. Like both AS and BBS, SARDS is marked by photoreceptor degeneration. Alterations in genes encoding structural ciliary proteins have been implicated in retinal degeneration noted in BBS and AS, as well as in the systemic pathologies associated with these syndromes. Functional loss of the *BBS8* gene (TTC8 protein) has been identified in Golden Retrievers with progressive retinal atrophy (PRA), concurrent with systemic symptoms such as weight gain and renal dysfunction, as well as a subjective assessment of anosmia (8). The expression of functional TTC8 is essential for the development of photoreceptor outer segments and for olfaction (9, 10). Furthermore, *BBS2* and *BBS4* genetic defects have been demonstrated in Shetland Sheepdogs and Hungarian Puli with PRA, respectively (11, 12). Both genetic defects yield a syndromic phenotype similar to those noted in human genetic ciliopathies, including retinopathy, polyphagia, weight gain, and renal dysfunction (3, 4, 11, 12). Interestingly, these heritable genetic defects associated with the ciliary BBSome protein complex do not always manifest early in canines compared to humans (8, 11, 12). While the authors acknowledge that PRA is a disease of retinal atrophy distinct from SARDS, we find it interesting that primary ciliary disorders have been implicated in canine retinal disease and hypothesize that an acquired ciliopathy in patients with SARDS could yield a phenotype similar to those observed with genetic defects. Ocular computed tomography (OCT) reveals similar findings in patients with BBS, AS, and SARDS, including disorganization/loss of photoreceptor outer segments and a blurred ellipsoid zone (13, 14). ERGs in patients with BBS and AS show greatly reduced amplitudes that often extinguish with time, while those in patients with SARDS are completely extinguished, by definition of the disease (2, 10). Vision loss associated with BBS and AS progresses at a moderate pace, with most patients becoming legally blind in the second decade of life (15, 16). Blindness in patients with SARDS occurs more rapidly, often within days or weeks, consistent with an acquired triggering event (2).

### 1.2 Appetite and hunger

Neurons associated with the regulation of appetite are primary ciliated cells, regulating hunger by binding adipocyte-released leptin to the requisite receptor associated with primary cilia. If these neuronal cilia are inhibited or lost, there is no feedback to the satiety centers from leptin signaling in the fed state, resulting in continuous feeding behavior. AS, BBS, and SARDS are marked by polyphagia with resultant weight gain (2–4). Patients with BBS and AS often have elevated plasma leptin concentrations due to the lack of feedback inhibition (17). However, the authors are unaware of leptin measurements taken in any patients with SARDS reported to date—such an evaluation could prove essential in understanding this syndrome. Polyphagia and obesity in AS and BBS can be treated by signal augmentation using setmelanotide (IMCIVREE™), which acts downstream of leptin binding. Administration of setmelanotide allows an exogenously administered ligand to bind to MC4R receptors located on secondary neurons (18). Reduction of weight gain in newly diagnosed patients with SARDS using setmelanotide could provide valuable insights not only into the pathogenesis of weight gain but also the cause of retinal degeneration. Weight loss in patients with SARDS following the use of setmelanotide would support the notion that this syndrome results from a systemic ciliopathy. Although GLP-1 agonists would likely treat polyphagia by prolonging gastric emptying time in both canine and human conditions of ciliopathy, such non-specific effects would not provide mechanistic insights into the cause of this syndrome (19).

### 1.3 Polyuria

The sensory function of the renal tubular primary cilium was described in Madine-Darby canine kidney (MDCK) cells over two decades ago (20). The state of fluid flow and osmolality in the tubular system in the kidney are assessed by primary cilia that project into the lumen of the tubule. Mechanical forces and electrolyte concentrations within the tubular lumen are surveyed by cilia, leading to adjustments in kidney function. In the Tg737 hypomorphic mutant mouse model, renal cilia are significantly shorter, and these animals are unable to concentrate urine (21). This phenotype is consistent with the shortened and dysfunctional primary cilia noted in patients with BBS and AS and their associated polyuria and impaired ability to concentrate urine. Patients with SARDS often exhibit polyuria with a low urine specific gravity, similar to that observed in patients with BBS and AS (2, 22, 23). Structural and biochemical evaluation of renal cilia in patients with SARDS may provide additional mechanistic insights into the cause of this syndrome.

### 1.4 Olfaction

Cilia associated with olfaction do not contain dynein and are, therefore, considered non-motile. Dogs with SARDS have a significantly diminished sense of smell compared to sighted or non-SARD blind dogs (1). In one study, using eugenol as an olfactory stimulant, the mean concentrations required to reach the sensory threshold were 0.017 g/mL, 1.70 × 10^−13^ g/mL, and 4.26 × 10^−13^ g/mL in dogs with SARDS, sighted dogs, and blind/non-SARDS dogs, respectively (1). Furthermore, genetic ablation of the *BBS8* gene in mice yields a profound loss of olfactory neurosensory cilia and a diminished sense of smell with systemic symptoms of retinal degeneration, obesity, and renal dysfunction (9). These genetic manipulations in mice may provide mechanistic insights into the loss of olfactory function observed clinically in dogs diagnosed with SARDS (1).

Patients with AS and BBS often have deficient olfaction (24).

## 2 Discussion

SARDS often presents with rapid loss of vision (days to weeks), associated weight gain, polyphagia, and often polyuria (2). Objective ophthalmic assessments include sluggish pupillary response to white light, no pupil response to red light, and a relatively normal response to blue light (2). A final diagnosis of SARDS is generally made based on the findings of an extinguished ERG (2). There are no specific or definitive clinical chemistries that can confirm a SARDS diagnosis.

The primary etiology of SARDS remains elusive. Historically, scientific inquiry has focused on an immunologic insult and/or endocrinopathy as the pathognomonic basis for SARDS. However, efforts to treat SARDS with immunosuppressants or modulators, such as corticosteroids, leflunomide, or IVIg, have been unsuccessful (2). Furthermore, efforts to identify anti-retinal antibodies in patients with SARDS have yielded conflicting results, with no definitive answers (2, 25). Clinicians have pointed to Cushingoid symptoms often observed in patients with SARDS, such as weight gain, increased appetite, and heightened thirst and urination. However, systemic cortisol concentrations pre- and post-ACTH stimulation typically do not differ between normal patients and those with SARDS (26). Nearly all of these Cushingoid symptoms can be readily produced by dysfunctional or absent primary cilia. Sudden loss or dysfunction of primary cilia would have a dramatic effect on vision since photoreceptors themselves are modified primary cilia. Without functional primary cilia, leptin cannot inhibit feeding by signaling satiety to secondary neurons. Polyuria noted in patients with SARDS could be secondary to the loss of primary renal cilia, leading to an inability to sufficiently concentrate urine. Hyposmia noted in patients with SARDS is also observed in human ciliopathies. Once vision loss and systemic symptoms manifest, they persist indefinitely in patients with AS, BBS, and SARDS (6, 7). The cause of vision loss, polyphagia, polyuria, and hyposmia noted in AS and BBS is known to result from ciliopathies, with an inability to sensor the local environment. Loss of function in the *BBS2*, *BBS4,* or *BBS8* genes and their requisite proteins has been identified in dogs with progressive retinal atrophy, concurrent with systemic symptoms such as anosmia, weight gain, and renal dysfunction (8, 11, 12). It seems prudent to carefully characterize genes associated with human ciliopathies in SARDS patients, examining alterations in sequence, epigenetic modification**s**, expression, and/or function. Although a genom**e**-wide association study (GWAS) was conducted on 15 dachshunds suffering from SARDS, no loci of interest were identified. This study was significantly underpowered to identify potential SNPs associated with SARDS (27). Therefore, the genetic basis for SARDS remains unanswered.

Loss of both vision and sense of smell may be particularly distressing for patients with SARDS. Prior to 2024, many clinicians reassured owners that the affected dogs would adapt and use their sense of smell as a primary means of navigation. However, it is now apparent that the majority of patients with SARDS also suffer significant olfactory dysfunction (1). Stuckey et al. indicated that the persistent systemic symptoms of polyphagia, subsequent weight gain, and polyuria “may represent a larger and more difficult management issue than blindness itself” (6). Systemic symptoms are prominent in both patients with BBS and SARDS and persist indefinitely (Table 1) (1, 2, 6, 23, 28). Agents that promote ciliary homeostasis and function, such as GPCR modulators, and/or stabilize ciliary structure, such as Tubastatin A and Aurora Kinase A inhibitors, or ciliary rescue therapies should be evaluated in clinical trials as potential therapeutic interventions (1, 29–31). Numerous drugs have been identified that may promote cilia regrowth, repair, and/or elongation in primary cancer cells in culture. However, these compounds were evaluated at concentrations (10 μM) well above those that are clinically relevant (32). Efforts to screen available drugs at lower, clinically relevant concentrations appear warranted. Investigating SARDS as a potential acquired ciliopathy could be a promising approach for seeking prevention or effective treatments.

**Table 1:** Prevalence of primary clinical symptoms associated with BBS and SARDS.

Clinical Symptom	BBS^a^	SARDS^b,c,d,e^
Retinopathy	94%	100%
Obesity/weight gain/polyphagia	89%	75%
Hyposmia	47–100%	**≈**100%
Renal dysfunction	52%	≤ 76.5%

^a^Forsyth et al. (28).

^b^Komáromy et al. (2).

^c^Leis et al. (23).

^d^Abrams et al. (1).

^e^Stuckey et al. (6).

## Data Availability

The original contributions presented in the study are included in the article/supplementary material, further inquiries can be directed to the corresponding author.
